# Visualizing the knowledge domains and research trends of childhood asthma: A scientometric analysis with CiteSpace

**DOI:** 10.3389/fped.2022.1019371

**Published:** 2022-09-30

**Authors:** Jinghua Wu, Yi Yu, Xinmeng Yao, Qinzhun Zhang, Qin Zhou, Weihong Tang, Xianglong Huang, Chengyin Ye

**Affiliations:** ^1^ Department of Health Management, School of Public Health, Hangzhou Normal University, Hangzhou, China; ^2^ Department of Epidemiology and Biostatistics, School of Public Health, Hangzhou Normal University, Hangzhou, China; ^3^ Department of Pediatrics, Zhejiang Provincial People's Hospital (Affiliated People's Hospital, Hangzhou Medical College), Hangzhou, China; ^4^ Department of Gastroenterology, Hangzhou Children’s Hospital, Hangzhou, China; ^5^ Department of Pediatrics, Xihu District Hospital of Integrated Traditional Chinese and Western Medicine, Hangzhou, China

**Keywords:** childhood asthma, citespace, bibliometrics analysis, scientometric analysis, atopic march, asthma management

## Abstract

**Background:**

Asthma is one of the most common chronic diseases in children globally. In recent decades, advances have been made in understanding the mechanism, diagnosis, treatment and management for childhood asthma, but few studies have explored its knowledge structure and future interests comprehensively.

**Objective:**

This scientometric study aims to understand the research status and emerging trends of childhood asthma.

**Methods:**

CiteSpace (version 5.8.R3) was used to demonstrate national and institutional collaborations in childhood asthma, analyze research subjects and journal distribution, review research keywords and their clusters, as well as detect research bursts.

**Results:**

A total of 14,340 publications related to childhood asthma were extracted from Web of Science (core database) during January 2011 to December 2021. The results showed that academic activities of childhood asthma had increased steadily in the last decade. Most of the research was conducted by developed countries while China, as a developing country, was also actively engaged in this field. In addition to subjects of allergy and immunology, both public health aspects and ecological environmental impacts on the disease were emphasized recently in this research field. Keywords clustering analysis indicated that research on asthma management and atopy was constantly updated and became the two major research focuses recently, as a significant shift in research hotspots from etiology and diagnosis to atopic march and asthma management was identified. Subgroup analysis for childhood asthma management and atopy suggested that caregiver- or physician-based education and interventions were emerging directions for asthma management, and that asthma should be carefully studied in the context of atopy, together with other allergic diseases.

**Conclusions:**

This study presented a comprehensive and systematic overview of the research status of childhood asthma, provided clues to future research directions, and highlighted two significant research trends of asthma management and atopy in this field.

## Introduction

As a non-communicable disease that often onsets in childhood, asthma affects approximately 300 million people of all ages worldwide (1). Among children aged 0 to 18, asthma is the most common chronic disease and has been called the epidemic of the 21st century, ranking among the top 20 conditions worldwide for disability-adjusted life years (2). The ISAAC study estimated that the global prevalence of parent-reported and doctor-diagnosed asthma among 6-7-year-old children to be 10.8%, with lower rates in Northern and Eastern Europe (4.5%), and the highest rates in North America (20.0%) and Oceania (29.2%) (3). At the same time, asthma not only causes substantial disability, impaired quality of life, or deaths in children, but also brings significant health and economic burden to their families and the whole society. It was reported by the U.S. Center for Disease Control and Prevention that asthma was the third leading cause of hospitalization among children under the age of 15 years, and asthma attacks caused approximately 13.8 million days of absence in school-aged children in 2013 (4). Globally speaking, developed economies spent 1% to 2% of their healthcare budget on asthma (1). In England, 69% of parents or guardians of asthmatic children reported having to take time off work due to their children's asthma, and 13% had lost their jobs (5). In addition, it is worth noting that childhood asthma prevalence in developing countries is increasing rapidly with advancing industrialization and urbanization, with increased mortality related to asthma and higher burden observed in these underserved regions (6, 7). Therefore, new insights are demanded in order to develop localized asthma prevention and treatment strategies in developing countries, to reduce its morbidity and disease burden and to benefit the treatment of other related respiratory or allergic diseases.

Currently, emerging epidemiological studies of asthma have been reported and plenty of risk and protective factors have been identified and recognized. Evidence has shown that exposure to smoking, air pollution, obesity and viral infection etc. is associated with an increased risk for asthma, while vitamin D or fish oil supplementation and exposure to diverse environmental microbiome can decrease the risk of asthma or its symptoms (8–13). Meanwhile, progress has also been made in understanding pathophysiological mechanisms of asthma development and exacerbation in children, especially in cytokines, innate lymphoid cells, macrophages/monocytes, T cells, as well as epithelium (14–18). In terms of diagnosis, several scoring systems and novel measures of airway physiology (e.g., electronic nose breath prints) have been developed, in addition to traditional approaches based on symptoms and evidence of variable expiratory airflow limitation obtained from bronchodilator reversibility testing or other tests (19–21). In terms of treatment and management, although treatments for type 2 (T2) inflammations, including drugs targeting interleukin (IL)-4, IL-5, and IL-13, have been well developed over the past decade, population heterogeneity and medication adherence are still challenges and broad psychosocial, behavioral or public health approaches should be further explored (22). The Global Initiative for Asthma (GINA) Strategy Report points out that asthma management is not a “one-size-fits-all” solution but a personalized assess–treat–review cycle, where either regular personalized assessment, treatment of modifiable risk factors, self-management education, skills training, or appropriate medication adjustment and review are essential to optimize treatment outcomes (19). On the other hand, the concept of atopic march has recently been put forward, referring to the natural progress of allergic diseases. This atopic march usually develops throughout the course of infancy and childhood, begins with atopic dermatitis (AD), and progresses to IgE-mediated food allergy (FA), asthma, and allergic rhinitis (AR). This well-recognized concept has also provided a new perspective for mechanistic research, prevention, treatment and management of asthma (23).

Although studies on asthma have increased substantially, researchers have not systematically analyzed the evolution of academic achievements in this field over the last decades. Therefore, a literature review is needed to understand the research status and future interests of childhood asthma. Scientometric measures and analyzes the quantitative features and characteristics of science, science communication, and scientific research (24). As a component of scientometric analysis, bibliometric mapping/analysis combines mathematical and statistical methods of quantitative analysis and has been widely used in various fields to describe academic landscapes, explore evolutionary processes and predict emerging trends in a particular study field (25, 26). CiteSpace, an informational visualization software which was developed by Chaomei Chen using Java programming language, can combine information visualization methods, bibliometrics, and data mining algorithms in an interactive visualization tool to extract patterns in citation data (27). This software has been widely used to conduct scientometric analyses in various scientific areas, such as harnessing telemedicine, Alzheimer's disease, HIV and so on (25, 28, 29).

However, limited study has been conducted to analyze bibliometric and scientometric features of scientific studies related to childhood asthma, and to explore the research trends and hotspots of this field. In this study, we aimed to employ CiteSpace to analyze the retrieved literatures in this research field in a visualization way, constructing co-occurrence networks and detecting the research hotspots and frontiers by time. It is anticipated that this work could provide a comprehensive and systematic understanding of recent research status and give clues to the development trends of childhood asthma.

## Materials and methods

### Data source

**Figure 1:**
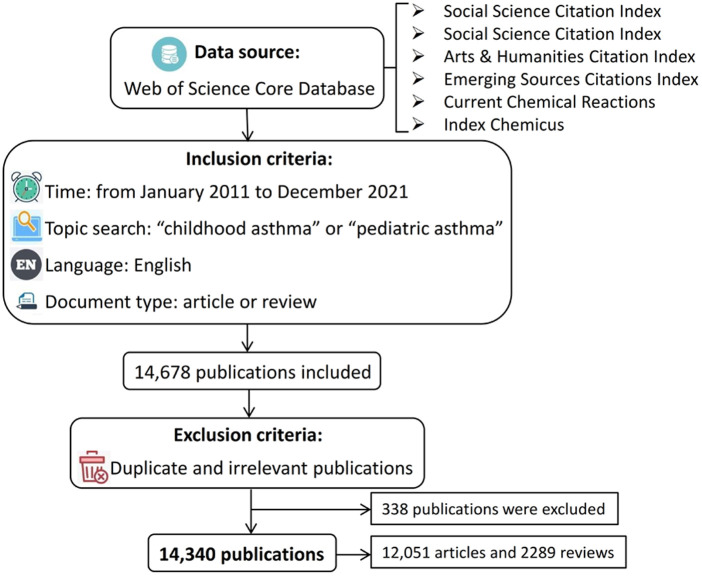
Workflow of data selection.

First, Web of Science (WOS) core database, which includes Social Science Citation Index (SCI-EXPANDED), Social Science Citation Index (SSCI), Arts / Humanities Citation Index (AHCI), Emerging Sources Citations Index (ESCI), Current Chemical Reactions (CCR-EXPANDED) and Index Chemicus (CI), were used as the data source to conduct an academic search. During the retrieval process, the inclusion criteria for publications were as follows: (1) published between January 2011 and December 2021, (2) the topic search was “childhood asthma” or “pediatric asthma”, (3) published in English, (4) the document type was “article” or “review”. Initially, a total of 14,678 publications were collected. Then, by using CiteSpace, 338 publications were excluded due to duplication or irrelevance. Finally, a total of 14,340 literatures, containing 12,051 articles and 2,289 reviews, were collected and used for the following analysis. More details about the workflow of data collection could be found in Figure 1. Bibliographic records of all these studies were downloaded, including title, abstract, author details, affiliation, keywords, and citations.

In subgroup keywords analysis, those met all of the following criteria were included in data of “childhood asthma management”: (1) published between January 2011 and December 2021, (2) the topic search was “childhood asthma management” or “pediatric asthma management”, (3) published in English, (4) the document type was “article” or “review”. After excluding duplicate publications, 2,765 literatures with their bibliographic records were downloaded for analysis. For the subgroup analysis of atopy, the term “atopy” was used, and articles or reviews published between January 2011 and December 2021 in English were included. After excluding duplicate publications, a total of 4,770 literatures were collected.

### Tool profile

CiteSpace is a Java-based software developed by Chaomei Chen to map the structure and dynamics of knowledge domains in a certain scientific field *via* computer algorithms and interactive visualizations (30). Specifically, CiteSpace can be used to identify the leading collaborations among countries, authors, and institutions, visualize the distribution of research fields, analyze co-occurring keywords and burst terms, and generate clusters of keywords or co-citing reference publications (27). Compared with other methods such as co-citation, CiteSpace's keywords analysis also provided research focus and structure of a certain discipline, and is more suitable for analyzing the development trends and frontiers of either mature disciplines or emerging disciplines. In addition, CiteSpace's cluster analysis can categorize scientific papers and their features according to the degree of similarity, providing prominent groups, and discerning what methods and tools have been developed (27, 30).

### Data analysis

SPSS 24 was used to create the fitting curve for the number of publications. Journals' impact factors were all obtained from the 2020 version of Journal Citation Reports, which provides transparent, publisher-neutral data and statistics. CiteSpace (version 5.8.R3) was used for analyzing and visualizing co-occurrence and co-citation networks. Based on the collected data, we reviewed the main countries and institutional collaborations that contributed to childhood asthma research, analyzed the research categories and journals' distribution, summarized keywords and their clusters, and detected research bursts over the last decade. Besides, subgroup keywords analysis for childhood asthma management and atopy were also conducted respectively. First, to map and visualize networks, term source was set as title, abstract, author keywords and keywords plus, while node type was set as country, institution, category, cited journal or keyword, successively. In addition, we ran network analyses by using the cosine link reduction method and selected pathfinder to prune the merged network, as this technique can simplify the network and highlight the important structural features. Then, following the keyword co-occurrence analysis, we used the burst detection function to catch the decline or rise of a keyword and explored the emerging trend during a certain period of time. After that, to further study salient topics and research trends in childhood asthma, we conducted the cluster analysis, where similar objects were combined to determine related areas of study. Specifically, we employed the latent semantic indexing (LSI) algorithm to analyze adjacent terms and keywords of childhood asthma, and ultimately formed clusters. This algorithm can not only reflect the frequency of term occurrence but also overcome the situation of polysemy and synonym. The size of the cluster depends on the involved number of objects, as ID 0 (#0) is the largest cluster that contains the largest number of keywords. When measuring how similar an object was to its own cluster compared to other clusters, the silhouette value (S) was adopted, where S > 0.7 indicated a high level of term consistency within the certain cluster.

In terms of visualization, each item is presented as a node, and a link between two nodes describes co-occurrence or co-citation. A thicker link indicates that the two nodes cooperate or co-occur more often. Rings of different colors in the node represent the node's frequency of occurrence in different years (i.e., the larger the ring, the higher the frequency), and details about the year represented by each color can be found in the legend for each figure. It is worth noting that the betweenness centrality is an indicator that can be used to evaluate the importance of each item in the visualization network, generally items with centrality ≥0.1 will be displayed in purple in their outer circle, indicating that this node as a hub that has important interactive significance.

## Results

### Characteristics of publication results

#### Number of publications over the years

**Figure 2:**
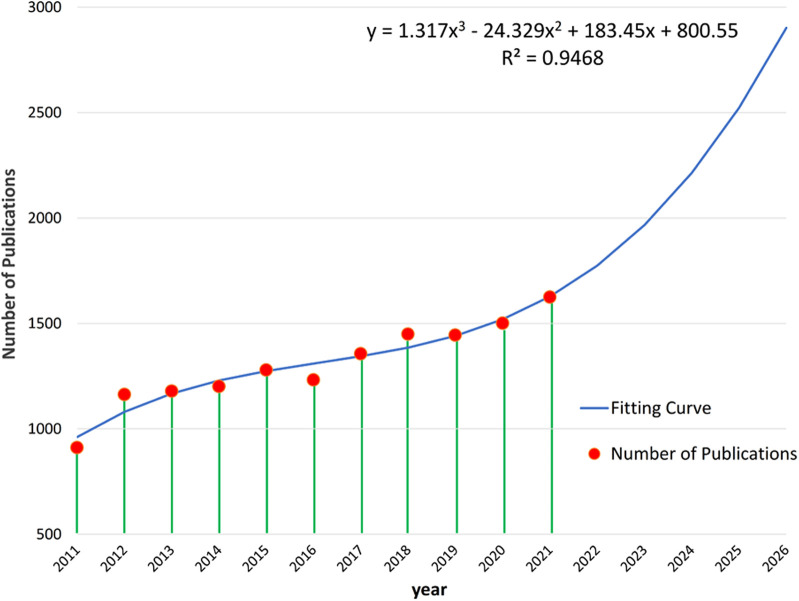
Yearly number of publications related to childhood asthma from 2011 to 2021.

A total of 14,340 publications related to childhood asthma were extracted from Web of Science (core database) during the last decade, from January 2011 to December 2021. In 2011, the publication number was 911, then, over the next decade, there was a steadily increasing trend in the number of publications in this research field. Besides, according to the cubic fitting curve (R^2 ^= 0.9468, *p* < 0.05), the number of publications was expected to continue growing rapidly over the next five years (Figure 2).

#### Subject trends and journal distribution in childhood asthma

**Figure 3:**
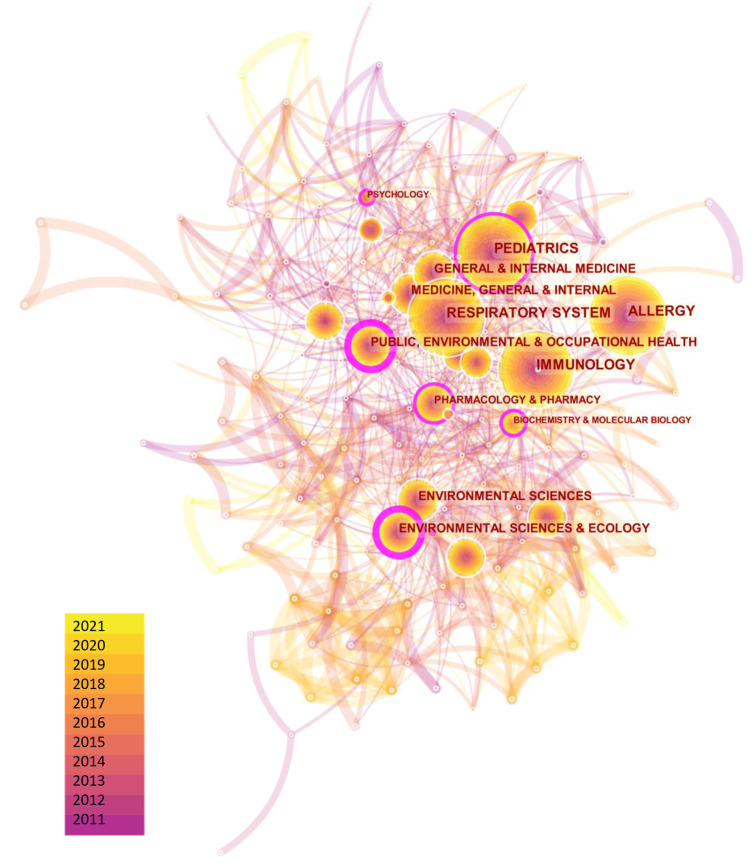
Distribution of subjects involved in childhood asthma research.

In order to explore the discipline distribution of the above 14,340 publications, we performed the co-occurrence analysis for their involved subjects. As a result, a total of 182 nodes and 1,098 links were generated on the map, as shown in Figure 3. It was found that most publications centered on subjects of allergy, immunology, and pediatrics. Specifically, subjects (centrality > 0.1) with the most prominent collaborative influence on pediatric asthma were public, environmental and occupational health, environmental sciences / ecology and pediatrics (*Multimedia* Supplementary Appendix 1), showing a trend of multi-polarization of research subjects. When analyzing the engaged journals in the research field, the top three co-cited journals were sorted as follows: J ALLERGY CLIN IMMUN (9,375, IF2020 = 10.793), AM J RESP CRIT CARE (6,958, IF2020 = 20.405) and EUR RESPIR J (6,342, IF2020 = 16.671). These journals were also the hubs in the network having relatively more interactions with other journals, indicating their significant impacts on childhood asthma research (see *Multimedia* Supplementary Appendix 2). It was worth noting that, two comprehensive top-level medical journals, “NEW ENGL J MED” and “LANCET”, were also found to provide research frontiers and hotspots in childhood asthma, facilitating the construction of the disease's knowledge base from perspectives of medicine, biology, management and psychology.

#### Regional and institutional distribution in childhood asthma

**Figure 4:**
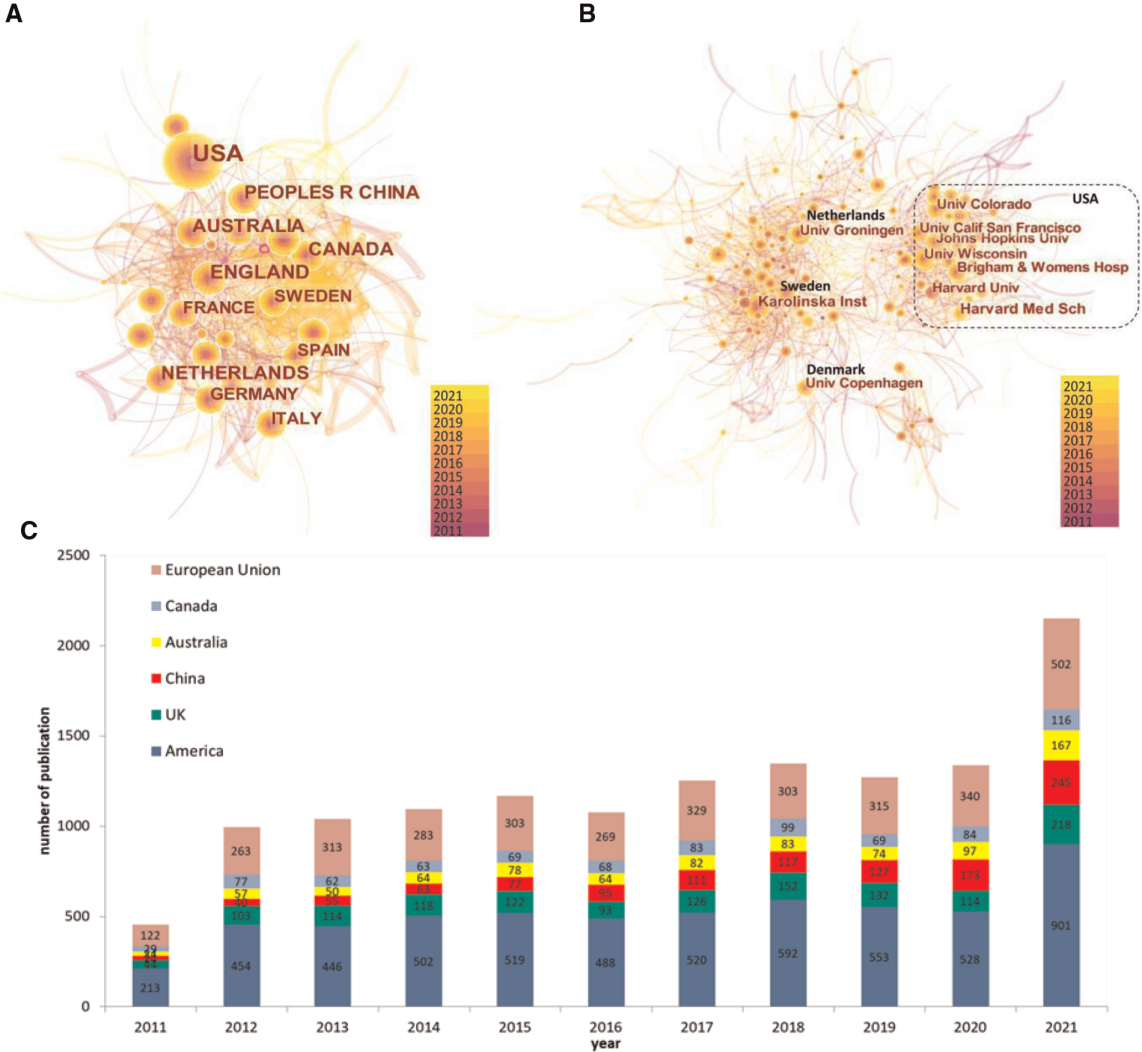
Distribution of (**A**) regions, (**B**) institutions, and (**C**) the top-10 countries' annual number of publications in childhood asthma research. *European Union includes Netherlands, German, Italy, Sweden and Spain.

We also generated regional and institutional distribution maps using CiteSpace to explore the contribution of different countries and the collaboration among major institutions to childhood asthma research. Figure 4A showed the collaborative network between different countries, where 144 nodes and 1,679 links were generated in total. It was found that, most of the top 10 countries actively in this field were concentrated in developed countries, including the USA (*n* = 5,716), the UK (*n* = 1,336), or European Union countries (*n* = 3,342), etc. On the other hand, as a developing country, People's Republic of China ranked third in the number of publications and it surpassed the UK since 2019, indicating that, along with the rapid development of the economy and technology, research interests in childhood asthma in China have a booming trend (Figure 4C), which may be attributed to the continued increase in asthma prevalence in this country. However, no country's centrality was ≥0.1, indicating that cooperation among countries has yet to be promoted. The distribution of institutions engaged in childhood asthma has been displayed in Figure 4B. Geographical location had a great impact on scientific collaboration between institutions, as the network revealed that the institutions with active collaborations were mainly divided into two parts, one was gathered by universities and hospitals in the USA, and the other is assembled by European countries in a relatively scattered way (see *Multimedia* Supplementary Appendix 3).

### Knowledge domains and emerging trends of childhood asthma

#### Keywords co-occurrence and cluster analysis in childhood asthma

**Figure 5:**
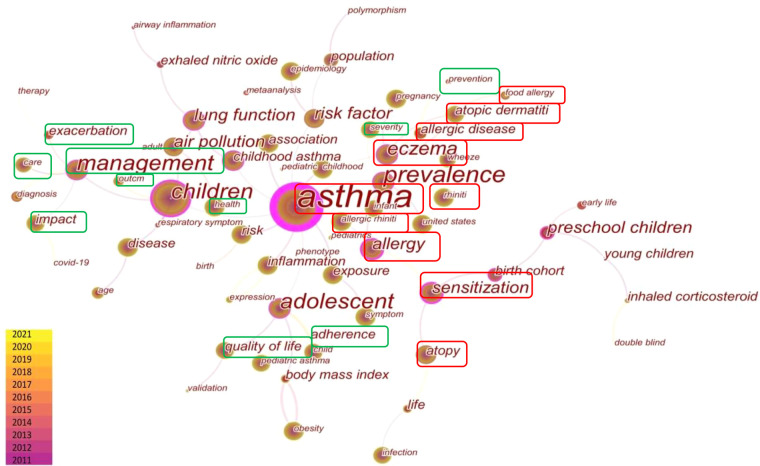
Distribution of co-occurring keywords.

Keywords reflect the core and focus of a paper. A total of 76 keywords were identified in the field of childhood asthma during the keyword co-occurrence analysis, revealing the research topics of greatest interests (Figure 5). Then, we classified the top keywords into 6 different themes to identify research priorities (Table 1), including atopy, management, risk factor, population, pathogenesis and diagnosis. Among them, the top 10 keywords ranked by frequency and centrality were also listed in the *Multimedia* Supplementary Appendix 4. Besides, we further conducted the cluster analysis by LSI algorithm to aggregate closely related and often concurrent keywords together, detailed information was summarized in Table 2 and *Multimedia* Supplementary Appendix 5. A total of 9 clusters were obtained and their silhouette values were all greater than 0.8, indicating that the keywords under each cluster attained a high level of consistency.

**Table 1:** Research keywords in childhood asthma listed by theme.

Theme	Most frequent keywords
Atopy	Asthma, allergy, atopy, atopic dermatitis, sensitization, eczema, allergic rhinitis, rhinitis, food allergy, allergic disease
Management	Health, management, quality of life, prevention, adherence, exacerbation, care, impact, outcome, severity
Risk factor	Risk, risk factor, exposure, air pollution, obesity, body mass index, epidemiology
Population	Children, childhood, preschool children, birth cohort, young children, early life, adult, infant, population
Pathogenesis	Inflammation, airway inflammation, polymorphism, phenotype, expression
Diagnosis	Symptom, wheeze, lung function, exhaled nitric oxide, respiratory symptom

**Table 2:** Detailed information for keywords clusters.

Cluster ID	Size	Silhouette	Year (mean)	Top Terms (LSI)
0	14	1	2012	*asthma*; childhood; symptom; mechanism; glutathione
1	11	0.962	2012	*children*; severity; association; eczema; sensitization
2	10	0.947	2012	*air pollution*; risk factors; chronic obstructive pulmonary disease; cigarette smoking; pm2.5
3	10	0.983	2012	asthma; *obesity*; atopy; physical education; smartphone
4	7	0.978	2012	*atopic dermatitis*; management questionnaire; Bacillus Calmette Guerin; supplementation; regulatory T cells
5	7	1	2012	*preschool children*; allergic rhinitis; otitis media; respiratory virus; milk sensitization
6	6	0.974	2012	*allergic rhinitis*; cohort study; disease prediction; sleep disturbances; learning disability
7	6	0.8	2011	asthma; *allergy*; atopy; eczema; microbiome
8	5	0.957	2011	asthma; *risk factor*; epidemiology; eczema; childhood

Specifically, the most predominant theme was atopy. Atopic march usually begins in early life, and the most common feature of these diseases is allergy (cluster#7) or sensitization. Poor control of one allergic disease often triggers or aggravates others, and thus having atopic dermatitis (cluster#4) or food allergy in early life greatly increases the risk of allergic rhinitis (cluster#6) and asthma (cluster#0) later. Therefore, asthma, as an end point event of atopic march, is often placed in the context of atopy and co-studied with other allergic diseases. Another extensive theme is childhood asthma management, the keywords indicated that, according to the severity of asthma, a series of care services were investigated and explored to improve patients' adherence, prevent exacerbation of asthma, and ultimately achieve the outcome of improving the quality of life and health status for children.

In the theme of risk factors, exposure to air pollution (cluster#2) and obesity (cluster#3) were the most commonly studied factors. Researchers also focused on the impact of cigarette smoking and PM2.5 on asthma and the risk of developing COPD in the future. Physical education *via* smartphones was considered as an important way to manage obesity in asthmatic children. As for population, preschool children (cluster#5) received more attention, with particular research focus on the disease “severity” and “association” with atopy, such as allergic rhinitis and milk sensitization. Lastly, inflammation and polymorphism has been widely investigated in the pathogenesis theme, and combining respiratory symptoms and exhaled nitric oxide to access lung function was the continuous focus in the asthma diagnosis theme.

#### Keyword burst detection in childhood asthma

**Figure 6:**
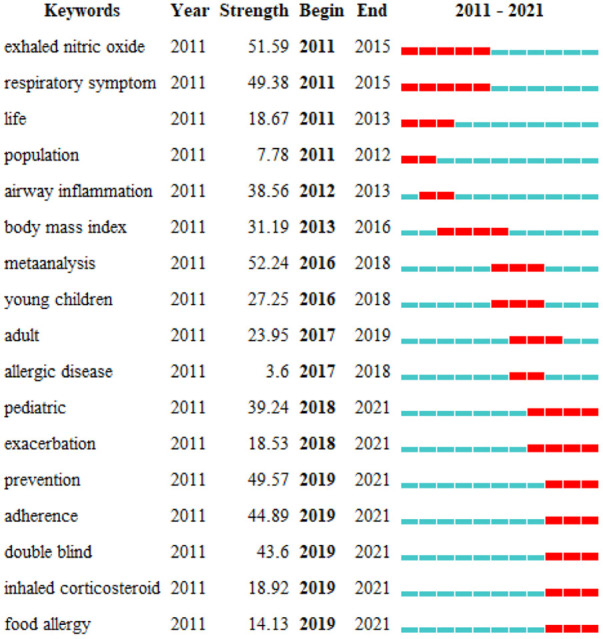
Top 17 keywords with the strongest citation bursts.

To further investigate changes of research hotspots over the last decade, we used the CiteSpace built-in burst detection algorithm and detected 17 keywords that had citation bursts (Figure 6). The burst strength was calculated as the growth rate of keywords being cited over time. Notably, the result showed that research hotspots have shifted significantly over the last decade.

#### Landmark publications from 2011 to 2016

From 2011 to 2016, the research hotspots were mainly related to the categories of asthma etiology (e.g., mechanisms of airway inflammation), diagnosis (e.g., exhaled nitric oxide detection and respiratory symptoms), and risk factors (e.g., obesity related body mass index), where most of the publications were related to development of diagnostic/prognostic tools or investigation of risk factor (Table 3). To facilitate asthma prognostic, Leif Bjermer et al. demonstrated that exhaled nitric oxide was a valuable tool for improving the personalized management (31). With regard to airway inflammation, H L Petsky et al. provided evidence that monitoring airway inflammation by eosinophils in induced sputum was useful in reducing asthma exacerbations (35). Shu Mei Teo et al. proposed that early asymptomatic colonization with streptococcus was a strong asthma predictor (33). In terms of risk factors, Guarnieri et al. showed that exposure to poor air quality may increase the risk of exacerbations of asthma symptoms (32). Notably, regarding to BMI, Pulgarón ER pointed out that the interaction between healthy weight and asthma control was still unclear (36).

**Table 3:** Lessons learned from landmark publications from 2011 to 2016.

Keyword	Author (year)	Citations	journal (2020IF)	Lesson learned
Exhaled nitric oxide	Bjermer et al. (2014) (31)	130	RESPIRATORY MEDICINE (3.415)	Fraction of exhaled nitric oxide is potentially a valuable tool for improving the personalized management of inflammatory airway diseases.
Respiratory symptoms	Guarnieri et al. (2014) (32)	899	LANCET (79.323)	Short-term exposures to ozone, nitrogen dioxide, sulfur dioxide, PM2·5, and traffic-related air pollution (TRAP) is thought to increase the risk of exacerbations of asthma symptoms.
Life	Teo et al. (2015) (33)	502	CELL HOST / MICROBE (21.023)	Asymptomatic colonization with streptococcus in early life is a strong asthma predictor.
Population	Fitzpatrick et al. (2011) (34)	302	J ALLERGY CLIN IMMUN (10.793)	Unique phenotypic clusters previously identified in adults can also be identified in children, but with important differences.
Airway inflammation	Petsky et al. (2010) (35)	1080	SCIENCE (47.728)	Monitoring airway inflammation through eosinophil in induced sputum is useful in reducing exacerbations, but it is debatable whether it should be universally advocated.
Body mass index	Pulgarón (2013) (36)	367	CLINICAL THERAPEUTICS (3.396)	It still needs to be determined whether a healthy weight helps regulate asthma symptoms and/or whether good asthma control helps maintain a healthy weight

#### Landmark publications from 2016 to 2021

From 2016 to 2021, the scholarly activity in childhood asthma was shifted to themes of asthma management and atopy, which were different from the earlier landmarks (Table 4). With regard to asthma management, how to improve adherence and prevent exacerbation gradually attracted more attention recently. Specifically, Petsky et al. presented a method of reducing asthma exacerbation by adjusting treatment to sputum eosinophil (35). Greer et al. reported that breastfeeding ≥3 to 4 months can prevent wheezing in the first 2 years of life and a longer duration of breastfeeding can prevent asthma even after 5 years of age (42). Trivedi et al. reported poor adherence with inhaled glucocorticoids (41). In order to overcome this problem, Gupta et al. introduced a sensor-based inhaler monitoring with clinical feedback intervention to potentially improve the adherence of inhalable corticosteroids (43). From the perspective of atopy, allergic diseases, especially food allergy, had showed the citation burst. Lodge et al. described that breastfeeding had varying degrees of protection against various allergic diseases (40), and Peters et al. reported that the prevalence of allergic diseases, including asthma, has risen sharply (45).

**Table 4:** Lessons learned from landmark publications from 2016 to 2021.

Keyword	Author (year)	Citations	Journal (2020IF)	Lesson learned
Mata analysis	Castro-Rodriguez et al. (2016) (37)	129	J ALLER CLIMM-PRACT (8.861)	Parental asthma, prenatal environmental tobacco smoke and prematurity are well-established risk factors for childhood asthma. Current findings do suggest mild to moderate causal effects of certain modifiable behaviors or exposures during pregnancy (maternal weight gain or obesity, maternal use of antibiotics or paracetamol, and maternal stress), the perinatal period (birth by Caesarean delivery), or postnatal life (severe RSV infection, overweight or obesity, indoor exposure to mold or fungi, and outdoor air pollution) on childhood asthma, but this suggestive evidence must be confirmed in interventional studies or well-designed prospective studies.
Young children	Dharmage et al. (2013) (38)	181	ALLERGY (13.146)	Young children with AD are at elevated risk of developing asthma.
Adult	Jolliffe et al. (2017) (39)	173	LANCET RESP MED (30.700)	Vitamin D supplementation safely reduces the rate of asthma exacerbations both in children and adults.
Allergic disease	Lodge et al. (2015) (40)	292	ACTA PAEDIATRICA (2.299)	Breastfeeding is protective for asthma (5–18 years). There is weaker evidence for a protective effect for eczema ≤2 years and allergic rhinitis ≤5 years of age, with greater protection for asthma and eczema in low-income countries.
Pediatric	Trivedi et al. (2019) (41)	57	FRONT PEDIATR (3.418)	Asthma varies considerably across the life course. Childhood asthma is known for high prevalence with a male predominance prior to puberty, common remission, and rare mortality. Childhood asthma severity is associated with duration of asthma symptoms, medication use, lung function, low socioeconomic status, racial/ethnic minorities, and a neutrophil phenotype.
Exacerbation	Petsky et al. (2012) (35)	81	THORAX (9.250)	Tailoring of asthma treatment based on sputum eosinophil is effective in decreasing asthma exacerbations.
Prevention	Greer et al. (2019) (42)	149	PEDIATRICS (7.125)	Any duration of breastfeeding ≥3 to 4 months can prevent wheezing in the first 2 years of life and a longer duration of any breastfeeding can prevent asthma even after 5 years of age.
Adherence	Gupta et al. (2021) (43)	6	PEDIATRICS (7.125)	The intervention of sensor based inhaler monitoring with clinical feedback has no influence on the adherence of inhalable corticosteroids in asthmatic children.
Double blind	Martineau et al. (2019) (44)	137	HEALTH TECHNOL ASSES (4.014)	Meta-analysis of those randomized, double-blind, placebo-controlled trials of supplementation with vitamin D-3 D-2 of any duration shows that vitamin D supplementation is safe and it protects against acute respiratory infections overall
Inhaled corticosteroids	Trivedi et al. (2019) (41)	270	THORAX (9.250)	Improving baseline asthma control with ICS can reduce the risk of exacerbations in patients with atopic asthma. But the overall adherence to ICS was approximately 50%, which was negatively correlated with the number of emergency department visits, fills of an oral steroid and the total days’ supply of oral steroid. Eight per cent of patients never filled their ICS prescription.
Food allergy	Peters et al. (2017) (45)	152	J ALLERGY CLIN IMMUN (8.861)	The prevalence of food allergy decreased between 1 and 4 years old but the prevalence of any allergic disease in Australia was remarkably high.

### Subgroup keywords analysis for childhood asthma management and atopy

Since “management” and “atopy” have become the research hotspots in childhood asthma in recent years, keyword subgroup analysis was further conducted on these two themes individually.

**Figure 7:**
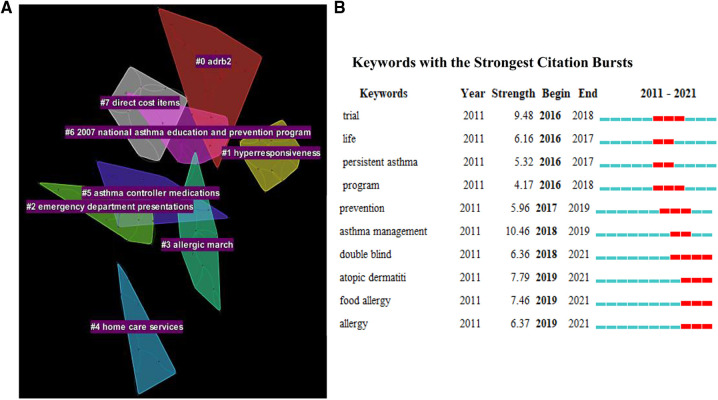
(A) Distribution of keywords cluster in childhood asthma management; (**B**) Keywords with the strongest citation burst nearly five years in childhood asthma management.

In the keyword cluster investigation for “childhood asthma management”, a total of 7 clusters were generated (Figure 7A). The most significant cluster was ADRB2 (cluster#0). The ADRB2 gene polymorphism was found to not only be associated with the susceptibility and severity of asthma, but also affected the therapeutic effect of bronchodilators, and thus specific genetic testing was recommended before certain medication treatments, so as to select the most suitable drug for pediatric asthma management (46, 47).

Cluster#1, #2 and #7 investigated the adverse effects of poor asthma management. Poor asthma control not only caused the hyperresponsiveness, but also increased direct and indirect cost, including hospitalizations, emergency department presentations, medical practitioner visits and medication. Meanwhile, clusters#3, #4, #5 and #6 attempted to provide solutions to minimize these adverse effects. First, plenty of studies suggested that asthma needs to be managed as part of the atopic march in association with other allergic diseases (cluster#3), which was consistent with our findings that atopy-related keywords (e.g., atopic dermatitis, food allergy and allergy) have recently shown the strongest citation bursts together with childhood asthma (Figure 7B). Second, due to the caregivers' cognitive errors, lack of knowledge, or difficulties in adhering to medication, researchers emphasized the family-based interventions, such as cognitive, behavioral and psychological education for caregivers (cluster#4) (48–50). With regard to medications, poor adherence to inhaled corticosteroids was still a key problem in asthma management, and global guidelines were constantly updated to adjust the dose and timing of medicines used in stepwise asthma management (cluster#5) (51–53). The “national asthma education and prevention program” was formed as cluster#6. The program, initiated by the National Heart, Lung, and Blood Institute in the United States, developed the first national consensus clinical practice guidelines for asthma treatment in 1991 and has been continuously updated since then. The program not only pointed out for the first time the significance of physician education, but also called for clinical education outreach, improved physician decision support tools, as well as redesign of health system, to facilitate childhood asthma management (54).

**Figure 8:**
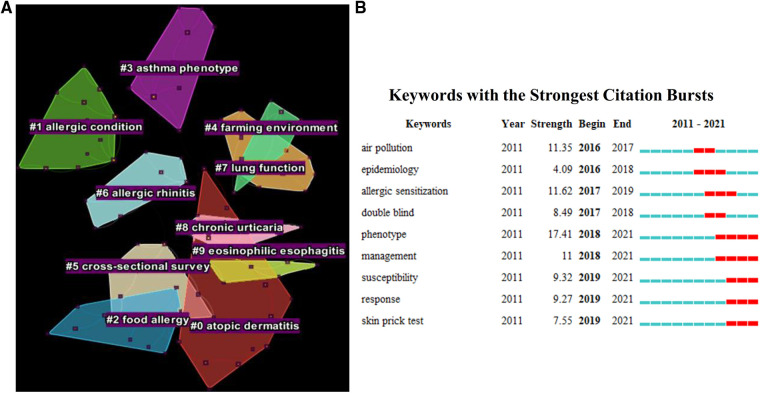
(**A**) Distribution of keywords cluster in atopy; (**B**) Keywords with the strongest citation burst nearly five years in atopy.

Similarly, cluster map and keywords analysis for the theme of atopy were performed and shown in Figure 8. A total of 10 clusters were captured, 7 of which were directly associated with atopy. During the course of the atopic march, atopic dermatitis (cluster#0), allergic condition (cluster#1), and food allergies (cluster#2) usually appear in infancy and then progress to asthma (cluster#3) or allergic rhinitis (cluster#6). All these allergic diseases formed independently as distinct clusters in our cluster map of the “atopy” theme. Besides, other gastrointestinal or skin symptoms or complications, such as chronic urticaria (cluster#8) and eosinophilic esophagitis (cluster#9), also appeared in the cluster map of atopy.

Notably, “asthma phenotypes” (cluster#3) not only formed a unique cluster, but also attained the strongest citation burst, indicating the significance of asthma phenotype research in the “atopy” theme. After retrieving the relevant literatures, it was found that heterogeneity of asthma was widely discussed recently. According to the atopic response, duration of asthma, and airflow restriction, childhood asthma could be divided into four phenotypes, namely late-onset symptomatic asthma, early-onset atopic asthma with normal lung function, early-onset atopic asthma with mild airflow limitation and co-morbidities, and early-onset atopic asthma with advanced airflow limitation (34). Another study of two large birth cohorts divided the typical symptom of asthma—wheeze into five phenotypes, which were never/infrequent wheeze, transient early wheeze, intermediate-onset wheeze, late-onset wheeze and persistent wheeze (55). Among them, intermediate-onset wheeze was found to be closely related to atopy, reduced lung function and increased airway hyperresponsiveness. It was worth noting that, in the keywords analysis of the “atopy” theme, “management” has also become an emerging hotspot since 2018, further suggesting that asthma control and prevention should be carried out in the context of atopic march.

## Discussion

### Summary

In this study, we searched the core data of WOS based on the search formula and obtained 14,340 literature data of childhood asthma from 2011 to 2021. Bibliometric and scientometric analysis was applied to explore the intellectual landscape and the evolution of trends. A sustained growth in the volume of publications was detected. The regional and institutional distribution network revealed that the USA and European countries were leaders in childhood asthma research, while China, as a developing country, was also emerging in this field. Keywords and cluster analysis showed that research on asthma management and atopy was constantly updated and became the research trends recently. The burst detection algorithm also found a significant shift of hotspots from etiology and diagnosis to atopic march and asthma management. In keywords subgroup analysis for childhood asthma management and atopy, it was found that asthma should be co-studied with other allergic disease while caregiver- or physician-based education should become important directions for improving medication adherence and asthma management.

### General distribution

Our analysis revealed a steady increase in publications in the field of childhood asthma, which was consistent with the rising trend of literatures reviewed by GINA report and other previous studies (56–58). In terms of regional output, the top 10 most prolific countries were almost all dominated by developed countries, which may be related to their advancement in industrialization. However, China, as a developing country, ranked third in terms of the number of publications and showed an increasing trend year by year. Moreover, in China, the prevalence of childhood asthma has raised significantly from 0.91% in 1990 to 2.12% in 2010, at an increasing rate of nearly 50% every 10 years (59). As one of the most populous countries with the fastest socio-economic development in the world, China's urbanization process has undergone unprecedented changes, resulting in dense populations moving into megacities and subsequent environmental challenges, such as air pollution (60). These rapid changes of lifestyle and environment may indirectly accelerate the increase of asthma prevalence and incidence, as explained by the immune system development theory of the hygiene hypothesis (61).

As an outcome of complex gene–environment interactions, asthma is usually triggered by environmental factors such as exposure to allergens. Research has also provided evidence of interactions among and between environmental and other intrinsic factors, such as genetics and atopy (62). As the prevalence of childhood asthma continues to rise, it is becoming a public health problem in both developed and developing countries. Therefore, with regard to subject categories, our study found that childhood asthma was not limited to the research of allergy, immunology and pediatrics, but is also widely studied from perspectives of public, environmental and occupational health, environmental sciences / ecology. Nowadays, activities conducted at the public health scale were incorporated into national guidelines to improve asthma outcomes, including asthma education and self-management training to increase patient adherence, coordination of care across various settings to reduce asthma triggers indoors and outdoors, and institutional reforms to improve quality of care (63–65).

### Emerging trends

We revealed two emerging trends in academic research on childhood asthma based on the keywords analysis. The first is that keywords such as atopic dermatitis (AD), allergic rhinitis (AR) and allergy appeared more frequently in the analysis results, indicating that the atopic march and asthma's relationship with other allergic diseases have been studied extensively. Epidemiological studies have provided strong evidence for the link between childhood asthma and other allergic diseases. A longitudinal study has found that early-onset AD (onset within first 2 years of life) increase the odds of developing childhood asthma (OR 1.74, 95% CI 1.30–2.34), and the presence of food allergy is also an independent risk factor of asthma (66, 67). This may be explained by the fact that allergic diseases in childhood shared common genetic, epigenetic and environmental risk factors, and their symptoms or underpinning pathogenesis is marked by disrupted skin, lung and gut barriers, altered microbiome and local and systemic Th-2-driven immunological pathways (68). Specifically, studies have shown that the occurrence of asthma, bronchial, rhinitis, history of atopy, pollen allergy, and sensitization to food allergens is significantly higher in AD patients with IgE above 200 IU/ml compared with those who had IgE under 200 IU/ml (69, 70). At the molecular level, various asthma/atopy-related biomarkers from genomes, epigenetics, transcriptomes, and metabolomics have been gradually explored and evaluated for their role in more precise diagnosis and individualized therapy of allergic diseases (56, 71–74). A GWAS study has identified immune-related gene variants (e.g., FLG and GSDMB) shared by asthma, hay fever and AD, suggesting that these allergic diseases may share the same mechanism that leads to dysregulation of immune-related genes (75). Meanwhile, Qi et al. identified replicable DNA methylation sites (e.g., cg20372759 and cg08844313) as prediction biomarkers of asthma and rhinitis (76). For transcriptomes, Kord et al. revealed that miR-1 was the hallmark of allergic airway inflammation, which had the potential therapeutic value by directly inhibiting the eosinophilic response in patients with asthma or rhinitis (77). In addition, after metabolomic analysis of fecal and blood samples, as well as intestinal microbiome measures, Lee-Sarwar et al. found an inverse association of polyunsaturated fatty acids and other lipids with asthma at age of 3 years (78). Although several features of asthma and atopic march have been well recognized, its underlying mechanism remains widely debated. Therefore, more comprehensive and systematic studies of the interplay between genomic, epigenetic, transcriptomic, and metabolomic signatures, as well as their interaction with microbes, are still needed to better understand how AD may progress to asthma (56, 68, 79).

Secondly, asthma management was also considered as an emerging trend in this study, as keywords such as management, prevention, adherence and exacerbation have frequently occurred or showed bursts in more recent studies. Asthma is a chronic disease that is difficult to cure completely; therefore, management plays a pivotal role throughout the disease course, in order to achieve the goal of symptom control, and to minimize future asthma-related deaths, exacerbations, persistent airflow limitation and side-effects of treatments (80). On the other hand, asthma management may be affected by disparities in socioeconomic status and quality of health service, and thus a single universal management might not be suitable for all people (81). Additionally, the 2018 Global Asthma Report noted that many governments have overlooked asthma in their plans when addressing non-communicable diseases and have made little progress in improving access to asthma management (82). Therefore, numerous studies are emerging to explore efficient strategies for asthma management, such as monitoring and assessing the effectiveness and outcomes of asthma management activities at the global and national levels, or exploring personalized disease management interventions at the individual level (81, 82). Eakin et al. improved asthma control and reduced the course of oral corticosteroids therapy and length of hospital stay in children through an intervention that combined family asthma education and a federally funded Head Start program (83). In addition, research has recently been emerging on Internet-based solutions, social media, and mobile technologies to improve asthma self-management in children and to facilitate the delivery of patient care (84–86). Wiecha et al. suggested that a multidimensional web-based educational, monitoring, and communication platform may have a positive impact on improving parental asthma-related knowledge and the use of asthma preventive medications in children (87).

### Recommendations for future study

Despite a continuous rise in scholarly activity in childhood asthma, we noticed several gaps in this study and put forward the following recommendations for future research accordingly. First, based on the fact that most of the institutional cooperation is carried out within a country, and the interactions between countries were still lacking, we advocate that academic institutes should strengthen collaborations and links among different countries, especially exchanges between developed and developing countries. Second, keyword analysis suggested that, in addition to these well-established asthma diagnostic techniques based on symptoms, exhaled nitric oxide, lung function, and detection of IgE immunoreactivity against specific allergens, potential biomarkers from multi-omics remain to be discovered to either assist the precise diagnosis/prediction of asthma or facilitate personalized treatment of the disease at the molecular level (74). Third, with the increasing understanding of the atopic march, identifying risk factors for allergic diseases should be considered crucial for early warning of childhood asthma. Moreover, studies are needed to explore the role of age, severity, phenotype heterogeneity, and genetic characteristics to help better understand how AD develops into asthma, and to benefit disease prevention and management ultimately. Forth, pediatric allergy and asthma still face challenges in seeking appropriate care and management currently, so more efforts should be made to improve asthma control, prevent asthma attacks, and reduce asthma burden (7). As reported by CDC of USA, a total of 50.3% of children with current asthma had uncontrolled asthma during 2012–2014, thus, by integrating various medical, behavioral, and psychological theories and research findings, large intervention trials are still required to explore better disease management strategies to improve patients' adherence and prevent asthma exacerbations (88). Lastly, with the current outbreak of coronavirus disease-2019 (COVID-19), questions about the link between childhood asthma and COVID-19 remains unclear. (i.e., does asthma contribute as a risk factor of COVID-19 infection? Are asthma medications protective or detrimental for COVID-19 infection?). Given the limited number of pediatric cases of COVID-19 worldwide, international collaborations are badly needed to illuminate this field.

### Limitations

There are some limitations in this study. First, we only collected data from the Web of Science Core Collection Database when retrieving literatures, while future research could be extended to other databases. Second, our study only extracted data on childhood asthma from 2011 to 2021, and thus we have only reviewed the research trend of childhood asthma over the last decade. Third, due to the software's features and limitations, the information analysis by CiteSpace was not based on the full text, and also the visualization of figures may lack some crucial details.

## Conclusion

In conclusion, based on the WOS core database, we adopted the analysis tool CiteSpace and obtained a systematic and comprehensive overview of childhood asthma research from 2011 to 2021, which provided important insights into research priorities, trending themes, and future directions. The number of childhood asthma studies has shown an ascending trend over the past decade. Developed countries and their institutions played a leading role in this research field, while developing countries such as China also actively engaged in this field. Furthermore, the management and prevention of childhood asthma, as well as the pathogenesis of atopic march, were considered two emerging trends recently. We hope this systematic bibliometric analysis will help discuss future research directions, provide effective strategies for interdisciplinary research, as well as promote cross-regional collaborations in childhood asthma studies.

## Data Availability

The original contributions presented in the study are included in the article/Supplementary Material, further inquiries can be directed to the corresponding author/s.
