# Supplementary material for: Visualizing the knowledge domains and research trends of childhood asthma: A scientometric analysis with CiteSpace
Source: Front Pediatr. 2022 Sep 30;10:1019371. doi: 10.3389/fped.2022.1019371 (PMC9562269; doi:10.3389/fped.2022.1019371)
Supplement: Supplementary file 1 [file Table1.docx]

Supplementary Table 1. Summary of the most common or important subjects related to childhood asthma research

| Rank | Frequency | Subjects | Centrality | Subjects |
| --- | --- | --- | --- | --- |
| 1 | 3618 | allergy | 0.37 | public, environmental and occupational health |
| 2 | 3268 | immunology | 0.21 | environmental sciences & ecology |
| 3 | 2737 | pediatrics | 0.17 | engineering |
| 4 | 2403 | respiratory system | 0.16 | pediatrics |
| 5 | 1271 | public, environmental and occupational health | 0.15 | pharmacology & pharmacy |
| 6 | 1129 | General & internal medicine | 0.14 | biochemistry & molecular biology |
| 7 | 752 | medicine, general & internal | 0.11 | psychology |
| 8 | 677 | environmental sciences & ecology | | |
| 9 | 666 | environmental sciences | | |
| 10 | 450 | science & technology-other topics | | |
