# Supplementary material for: Visualizing the knowledge domains and research trends of childhood asthma: A scientometric analysis with CiteSpace
Source: Front Pediatr. 2022 Sep 30;10:1019371. doi: 10.3389/fped.2022.1019371 (PMC9562269; doi:10.3389/fped.2022.1019371)
Supplement: Supplementary file 2 [file Table2.docx]

Supplementary Table 2.Summary of the most co-cited or important journals

| Rank | Frequency | Journal | Centrality | Journal |
| --- | --- | --- | --- | --- |
| 1 | 9375 | J ALLERGY CLIN IMMUN | 0.86 | J ALLERGY CLIN IMMUN |
| 2 | 6958 | AM J RESP CRIT CARE | 0.62 | AM J RESP CRIT CARE |
| 3 | 6342 | EUR RESPIR J | 0.35 | EUR RESPIR J |
| 4 | 6200 | PEDIATRICS | 0.34 | J IMMUNOL |
| 5 | 6021 | ALLERGY | 0.33 | THORAX |
| 6 | 5344 | CLIN EXP ALLERGY | 0.24 | PEDIATRICS |
| 7 | 5319 | NEW ENGL J MED | 0.23 | AM J EPIDEMIOL |
| 8 | 5278 | THORAX | 0.17 | NATURE |
| 9 | 5130 | LANCET | 0.16 | ENVIRON HEALTH PERSP |
| 10 | 4314 | J ASTHMA | 0.13 | ALLERGY |
