# Supplementary material for: Visualizing the knowledge domains and research trends of childhood asthma: A scientometric analysis with CiteSpace
Source: Front Pediatr. 2022 Sep 30;10:1019371. doi: 10.3389/fped.2022.1019371 (PMC9562269; doi:10.3389/fped.2022.1019371)
Supplement: Supplementary file 3 [file Table3.docx]

Supplementary Table 3.Top 10 most productive institutions

| rank | frequency | Centrality | institution | country |
| --- | --- | --- | --- | --- |
| 1 | 292 | 0.05 | Karolinska Inst | Sweden |
| 2 | 281 | 0.01 | Harvard Med Sch | USA |
| 3 | 231 | 0 | Harvard Univ | USA |
| 4 | 222 | 0.03 | Johns Hopkins Univ | USA |
| 5 | 219 | 0.03 | Univ Groningen | Netherlands |
| 6 | 218 | 0.02 | Univ Colorado | USA |
| 7 | 211 | 0.05 | Univ Calif San Francisco | USA |
| 8 | 208 | 0.02 | Univ Cppenhagen | Denmark |
| 9 | 201 | 0.02 | Univ Wisconsin | USA |
| 10 | 196 | 0.01 | Brigham & Womens Hosp | USA |
