# Supplementary material for: Visualizing the knowledge domains and research trends of childhood asthma: A scientometric analysis with CiteSpace
Source: Front Pediatr. 2022 Sep 30;10:1019371. doi: 10.3389/fped.2022.1019371 (PMC9562269; doi:10.3389/fped.2022.1019371)
Supplement: Supplementary file 4 [file Table4.docx]

Supplementary Table 4. The top 10 keywords in frequency and centrality

| Rank | Frequency | Keyword | Centrality | Keyword |
| --- | --- | --- | --- | --- |
| 1 | 7078 | asthma | 1.8 | asthma |
| 2 | 4944 | children | 0.54 | allergy |
| 3 | 3088 | childhood asthma | 0.52 | sensitization |
| 4 | 2027 | childhood | 0.49 | children |
| 5 | 1974 | prevalence | 0.45 | adolescent |
| 6 | 1651 | risk | 0.44 | childhood asthma |
| 7 | 1348 | association | 0.34 | birth cohort |
| 8 | 1242 | allergy | 0.31 | lung function |
| 9 | 1153 | risk factor | 0.31 | eczema |
| 10 | 1115 | exposure | 0.3 | preschool children |
