# Supplementary figures and images for: Visualizing the knowledge domains and research trends of childhood asthma: A scientometric analysis with CiteSpace
Source: Front Pediatr. 2022 Sep 30;10:1019371. doi: 10.3389/fped.2022.1019371 (PMC9562269; doi:10.3389/fped.2022.1019371)

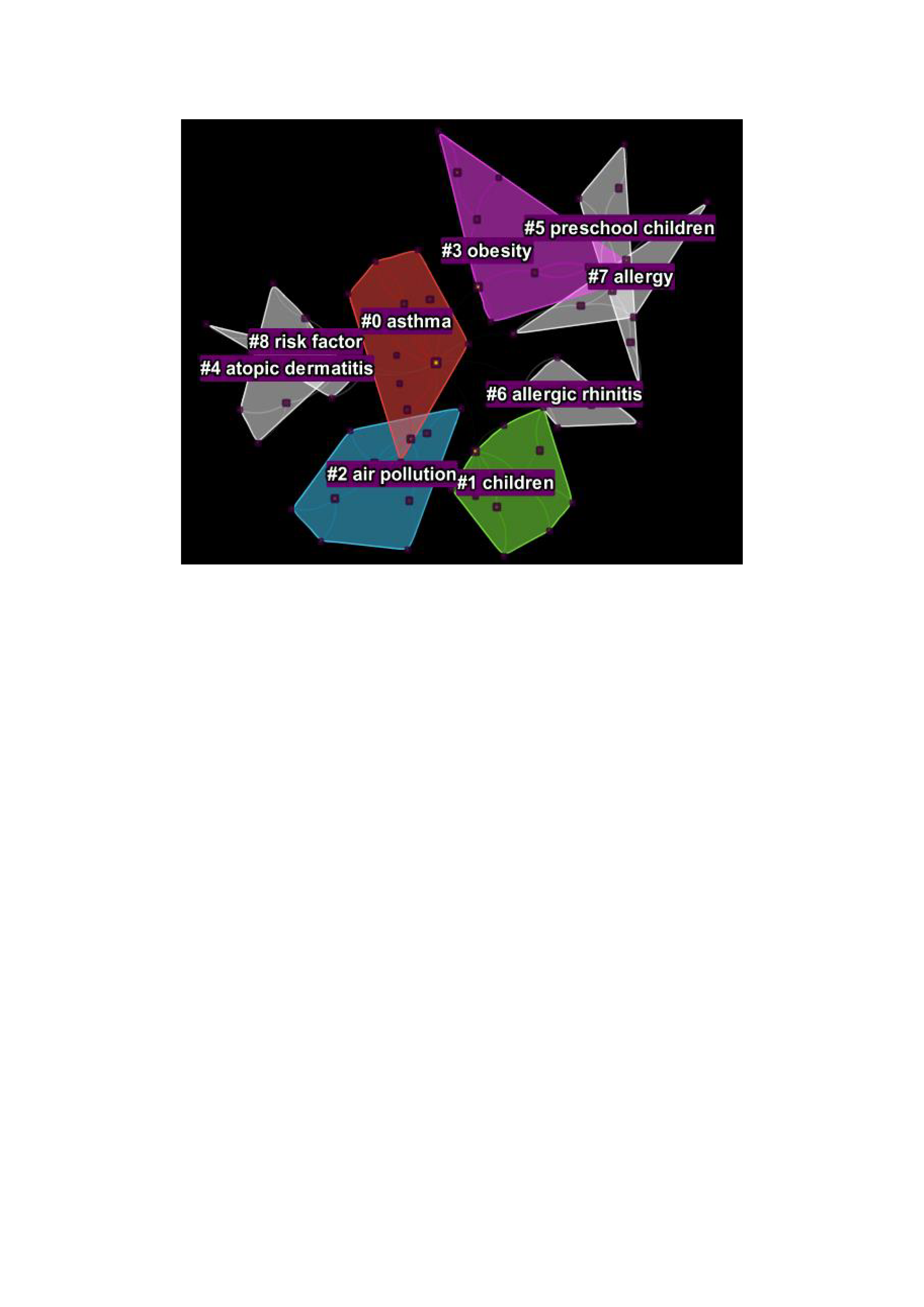

Supplement: Supplementary file 5 [file Image1.tif]
